# Working memory assessment using cambridge neuropsychological test automated battery can help in the diagnosis of mild cognitive impairment: a systematic review and meta-analysis

**DOI:** 10.1590/1980-5764-dn-2022-0006

**Published:** 2022-09-26

**Authors:** Zahra Sabahi, Mehdi Farhoudi, Amirreza Naseri, Mahnaz Talebi

**Affiliations:** 1 Tabriz University of Medical Sciences, Neurosciences Research Center, Tabriz, Iran.; 2 Tabriz University of Medical Sciences, Student Research Committee, Tabriz, Iran.; 3 Tabriz University of Medical Sciences, Research Center for Evidence-based-Medicine, Iranian EBM Center: A Joanna Briggs Institute Center of Excellence, Tabriz, Iran.

**Keywords:** Cognitive Dysfunction, Memory, Short-Term, Neuropsychological Tests, Systematic Review, Meta-Analysis, Disfunção Cognitiva, Memória de Curto Prazo, Testes Neuropsicológicos, Revisão Sistemática, Metanálise

## Abstract

Mild cognitive impairment (MCI) is an interstitial state between normal aging and dementia.

**Objective:**

In this study, we investigated working memory (WM) profiles of MCI patients using the Cambridge Neuropsychological Test Automated Battery (CANTAB). We also examined the diagnostic accuracy and possible associated factors as secondary outcomes of the study.

**Methods:**

We conducted an electronic search on EMBASE, PubMed, and ScienceDirect databases. Studies with MCI participants and using CANTAB battery subtests for the assessment of WM were included. Meta-analysis was conducted using the CMA2 software.

**Results:**

Out of 1537 records, 14 studies were covered in this systematic review, and 7 of them were included in the meta-analysis. There was a significant difference between MCI patients and healthy controls in spatial working memory (SWM) (SDM: 0.535; 95%CI 11–96; p-value=0.014), spatial span (SSP) (SDM: 0.649 95%CI 0.297–0.100; p-value<0.01), and rapid visual information processing (RVP) (SDM: 0.52; 95%CI 0.386–0.654; p-value<0.01). WM function of MCI patients was associated with the cerebrospinal fluid (CSF) levels of tau-protein and amyloid-beta (Aβ).

**Conclusions:**

WM is an impaired cognitive domain in MCI. CANTAB WM subtests including SSP, SWM, and RVP are accurate enough to be used as a proper assessment tool for the diagnosis of MCI in clinical settings. Tau-protein and Aβ are associated with lower WM scores in MCI patients; however, sex, age, psychiatric disorders, apolipoprotein 4 allele, and functional activity scores cannot affect WM.

## RESUMO

O comprometimento cognitivo leve (CCL) é um estado intersticial entre o envelhecimento normal e a demência.

**Objetivo:**

Neste estudo, investigamos os perfis de memória de trabalho (MT) de pacientes com CCL usando a bateria automatizada de testes neuropsicológicos de Cambridge (*Cambridge Neuropsychological Test Automated Battery –* CANTAB). Também examinamos a acurácia diagnóstica e possíveis fatores associados como desfechos secundários do estudo.

**Métodos:**

Foi realizada uma busca eletrônica nas bases de dados EMBASE, PubMed e ScienceDirect. Foram incluídos estudos com participantes com CCL e utilizando subtestes da bateria CANTAB para avaliação da MT. A meta-análise foi realizada usando o software CMA2.

**Resultados:**

Dos 1.537 registros, esta revisão sistemática abordou 14 estudos, e 7 deles foram incluídos na meta-análise. Houve uma diferença significativa entre pacientes com CCL e controles saudáveis na memória de trabalho espacial (MTE) (DPM: 0,535; IC95% 11–96; valor p=0,014), spatial span (SSP) (SDM: 0,649; IC95% 0,297–0,100; valor p<0,01) e processamento rápido de informação visual (PRV) (DPM: 0,52; IC95% 0,386–0,654; valor p<0,01). A MT de pacientes com CCL foi associada com os níveis de proteína tau e beta-amiloide (Aβ) no líquido cefalorraquidiano (CSF).

**Conclusões:**

A MT é um domínio cognitivo prejudicado no CCL. Os subtestes CANTAB WM, incluindo SSP, MTE e PRV, são precisos o suficiente para serem usados como uma ferramenta de avaliação adequada para o diagnóstico de CCL em ambientes clínicos. A proteína Tau e Aβ estão associadas a pontuações de MT mais baixas em pacientes com CCL; entretanto, sexo, idade, transtornos psiquiátricos, alelo da apolipoproteína 4 e escores de atividade funcional não podem afetar a MT.

## INTRODUCTION

Mild cognitive impairment (MCI) is known as a transitional state between normal aging and dementia in the age continuum in which patients experience memory loss more than healthy age-matched older adults, but do not fulfill defined criteria for dementia diagnosis^1^. Based on manifestations and disease course, MCI includes different subtypes: amnestic or non-amnestic MCI and single- or multiple-domain MCI^2^. The amnestic MCI is typically associated with an increased risk of conversion to Alzheimer's disease (AD); however, non-amnestic subtypes, which may progress to non-AD dementias, may also evolve to AD^3^.

Several studies have estimated the prevalence of MCI from 12 to 18% in older people over the age of 60 years^4–8^. With the global increase in life expectancy, early diagnosis and precise application of disease-modifying treatments for MCI have turned into a priority for the health systems^9,10^. Previous studies following MCI patients for 6 years found that 80% of patients progress to AD with an annual rate of 10–15%^1,11^, which is 10-fold higher than the conversion rate in the normal population^12^.

Several cognitive domains such as learning, short- and long-term memory, social cognition, language, perceptual motor, complex attention, or executive functioning are characteristically affected by the pathogenesis of AD along with disease progression^13,14^. Working memory (WM) can be defined as a component of short-term memory with a restricted capacity that depends on central executive functions and attention, utilizing stored information and linking them to long-term memory^15^. Unlike short memory which provides short-term storage of information, WM has been proposed as a multicomponent structure that stores incoming information and operates them to a more complicated cognitive function^16–18^. WM is highly associated with daily functioning abilities^19^ and has shown an explicit linear decreasing relationship with age^20,21^ so it can be used as a measure for early diagnosis of dementia^22^.

Previous studies have shown impairment of WM in the early stage of dementia^23–26^, which makes it a good factor for early diagnosis of the disease and prevention of disease progression. Classic paper-pencil tests like Montreal Cognitive Assessment (MoCA) and Mini-Mental State Examination (MMSE) are widely being used for the assessment of MCI^27^; however, these tests have shown some serious drawbacks with standardization of administration, the accuracy of response measurement, and demographic factors, importantly years of education and illiteracy^28–31^.

The Cambridge Neuropsychological Test Automated Battery (CANTAB) is a computerized neuropsychological test with a game-like and non-verbal environment that assesses the different cognitive domains like memory, attention, executive functions, learning, and problem-solving^32^. Among various subtests of CANTAB, spatial span (SSP) and spatial working memory (SWM) account for the assessment of WM^33–35^. Also, rapid visual processing (RVP) accounts for sustained attention and target detection that has a small WM component that is sensitive to parietal and frontal lobe dysfunction^36^.

In this systematic review and meta-analysis study, we aimed to study the WM function in MCI patients using CANTAB to determine the severity of WM impairment in MCI patients, as the primary outcome, and compare it with healthy matched older adults, to define the diagnostic accuracy of WM profiles of CANTAB in the detection of MCI. Also, as another secondary outcome, we investigated the associated factors of WM function in MCI patients.

## METHODS

This study was conducted following the preferred reporting items for systematic reviews and meta-analyses (PRISMA) statement^37^. This systematic review was designed to assess the WM function in MCI patients using the CANTAB, and the meta-analysis was conducted to compare the differences between MCI and healthy participants in WM subtests of the CANTAB.

### Search

Two independent researchers (Z.S. and A.N.) conducted a systematic literature search on EMBASE, PubMed, and ScienceDirect databases combining the keywords “Cognitive dysfunction, cognitive decline, cognitive impairment, mental deteriorations, mild cognitive impairment, CANTAB, Cambridge Neuropsychological Test Automated Battery, neuropsychological test, working memory, immediate memory, short-term memory,” on December 16, 2020. For the sake of comprehensiveness, the references of each included study were checked for any additional related papers.

### Study selection

Search results were imported to the EndNote reference manager. After deleting duplicated studies, two independent authors (Z.S. and A.N.) started screening and selecting papers by title/abstract in the first stage and full text in the second stage. In case of any conflicts, investigators tried to convince each other or ask for a third expert researcher's comment (M.T. or M.F.).

Inclusion criteria were as follows:

1. Original journal articles,
2. MCI diagnosis at the baseline based on the clinical criteria,
3. Using CANTAB subtests that evaluate WM, and
4. Studies in English.

Exclusion criteria were as follows:

1. Studies in other languages, and
2. Other types of articles such as review articles, editorials, letters,
3. Conference abstracts, and
4. Animal studies.

### Data extraction

Data were extracted by two independent authors (Z.S. and A.N.) in a pre-specified format using a data extraction table, including the name of the first author of the study, publication year, study design, the overall number of participants as well as the number of patients in each group of the study, mean age, years of education, diagnostic criteria, MMSE score, mean and standard deviations (SD) of CANTAB WM subtests in MCI group and the healthy control group, and finally associated and non-associated factors with WM function. We could not examine amnesic and non-amnesic subtypes separately since they were not described in most of the included articles. The online version of Web Plot Digitizer was used for extracting the exact values from the graphs. Extracted data were reviewed by a third author (M.T. or M.F.) and, in case of any disagreements about results, it was determined between authors or by a judgment of a third author.

### Risk of bias in individual studies

The risk of bias (RoB) and methodological quality were evaluated (by Z.S. and A.N. separately) with Joanna Briggs Institute (JBI) checklist that contains eight questions, evaluating inclusion criteria, detailed study subjects and setting, the validity of exposure, the standard measurement of the condition, and the outcome, identifying and dealing with confounding factors and statistical analysis^38^.

### Statistics

In this study, meta-analysis was performed using comprehensive meta-analysis (CMA) version 2.0. The confidence interval was considered at 95% and 0.05 level of significance for the p-value. Studies that used SWM total errors, SSP length, as well as A’ or latency measures of RVP subtest of CANTAB in MCI patients and healthy control group were included in the quantitative analysis. The I^2^ model was also utilized for assessing the level of heterogeneity among included studies. Whenever any of the studies had reported data for MCI by subgroups (subjective MCI, amnestic MCI, single-domain MCI, multiple-domain MCI), we merged them using an excel code. The mean, SD, and the number of the individuals in each group were imported into CMA, and both the random-effect model (REM) and fixed-effect model (FEM) were utilized for assessing the difference between the groups. Also, the results of the study were reported in funnel plots in Supplementary Material.

## RESULTS

### Search results and selection process

**Figure 1:**
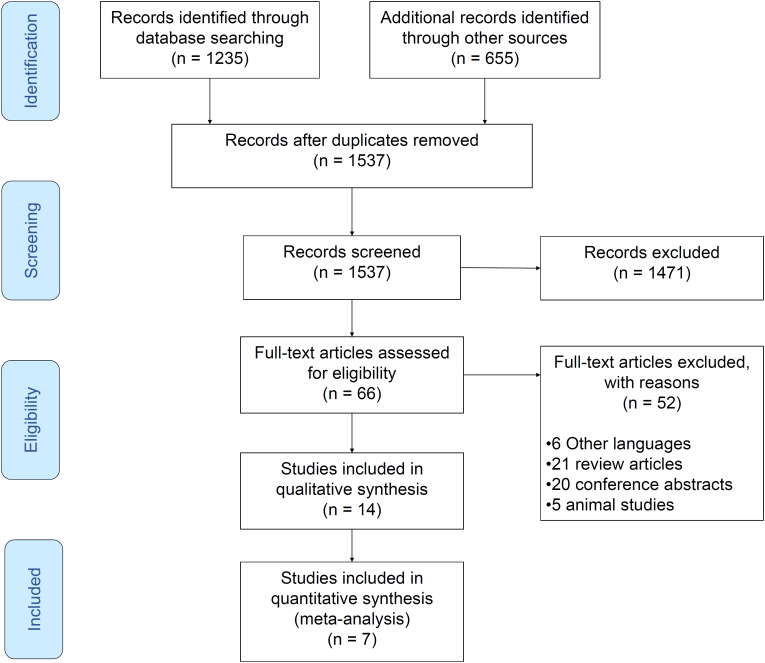
PRISMA flow diagram.

The electronic search identified 1,235 records through databases and 655 records added from other resources. After removing duplicates, 1,537 records were screened, and 1,434 records were excluded. Out of 66 studies that were assessed in the full-text stage, 14 studies were included in this systematic review, and 7 of them met our inclusion criteria for the meta-analysis. The PRISMA flow diagram is presented in Figure 1. Table 1 ^10,33,39–50^ is a summary of the characteristics and findings of included studies.

**Table 1:** Characteristics and summary of findings of included studies.

Author (year)	Study design	Sample size	Mean age	Education	Male ratio (%)	MMSE score	Eligibility criteria MCI	Eligibility criteria-control	CANTAB subtests for WM	MCI			Control			Associated factors	Non-associated factors
										Mean	SD	n	Mean	SD	n		
Nathan et al. 2017^40^	Cross-sectional	145	68.2±7.37	46.9% above 10 years of education	42.8	26.6±1.9 (24 to 30)	Age 55–90 years; subjective memory complaint verified by a family relative; at least 1 standard deviation deficit in a measure of episodic memory; an MMSE score of 24–30; a CDR scale score of 0.5 (with a score of 0.5 for the memory subscale); a clinical diagnosis of amnestic MCI, but preservation of general cognitive and functional performance to no meet clinical criteria for AD; GDS scale score <6; and Hachinski Modified Ischemic Scale score ≤4		SWM between errors	27.7	8.3	145				Aβ42, total tau, p-tau, hippocampal volume	Age, GDS, NPIQ, FAQ, MMSE, education, sex, APOE4
									SWM strategy	19.9	2.4						
									DMS % correct (all delays)	67.9	16.3						
									RVP A’	0.8	0.1						
Saunders and Summers et al. 2010^41^	Cross-sectional	131	Control: 69.32±5.83 S-MCI: 71.16±7.08 A-MCI: 71.18±7.02	Control: 13.56±3.14 S-MCI: 13.00±3.52 A-MCI:13.13±3.35	48.09	–	Memory problems, with a history of decline from a former level; preserved cognitive functioning; intact activities of daily living; no history of significant medical, neurological, or psychiatric condition; no history of major risk factors for vascular disease; and no history of alcohol abuse, sensory impairment, or impairment of hand mobility.	No cognitive complaints or medical history, participants with matched mean age and level of education	SWM total errors (S-MCI)	42.06	15.05	32	33.68	13.08	25	Age, WTAR (a-MCI), DRS II (a-MCI), BNT (a-MCI), RAVLT trial 5 recall (a-MCI), RAVLT delayed (a-MCI)	Education, sex, WTAR (S-MCI), WAIS-II FSIQ, GDS, PAL, DRS II (S-MCI), BNT (S-MCI), RAVLT trial 5 recall (S-MCI), RAVLT delayed (S-MCI)
									SWM total errors (A-MCI)	51.82	15.15	60					
									SSP length (S-MCI)	5.20	0.69	32	5.80	0.76			
									SSP length (A-MCI)	4.96	0.64	60					
									RVP A’ (S-MCI)	0.86	0.05	32	0.93	0.04			
									RVP A’ (A-MCI)	0.87	0.05	6					
									RVP latency (S-MCI)	530.70	123.75	32	447.50	77.51			
									RVP latency (A-MCI)	519.68	123.57	60					
Egerházi, et al. 2017^42^	Cross sectional	40	55±6	–	47.5	28±0.6	No neurological symptoms or other physical disorders, amnestic MCI diagnosis according to the criteria of Petersen, CDR= 0.5, mild short-term memory loss, with symptoms insufficient for the diagnosis of dementia according to the criteria of the DSM-IV, MMSE≥26, normal CT/MRI, No medication intake		SWM (Z-score)	-0.8871		25	–	–	–	–	–
									SSP (Z-score)	-0.755			–	–	–		
									RVP (Z-score)	-2.101			–	–	–		
Collie et al. 2002^39^	cohort	46	Control: 65.94±5.37 MCI: 67.82±7.75	Control: 12.37 ±3.78 MCI: 11.64±3.86	45.65	28.12±1.42	Age >50 years, no psychiatric and neurological diagnosis. Exclusion criteria at this stage included a history of respiratory, circulatory, or endocrine disease, personal or family history of psychiatric illness, head injury or substance abuse. MCI based on CERAD neuropsychological battery		SWM Total errors	33.33	15.81	23	25.19	12.24	23	CERAD (word list recall and learning)	Age, sex, education, MMSE, depression, CERAD, APOE4, CFQ, WMS-R, state and trait anxiety test, and NART
									SWM Strategy score	17.04	3.24	23	15.15	2.57	23		
									SSP task score	4.74	1.05	23	5.38	0.90	23		
Facal et al. 2014^42^	Cross sectional	145	MDA-MCI: 70.34±9.49 SDA-MCI: 67.62±9.40 Control: 67.36±9.09	MDA-MCI: 9.54±3.77 SDA-MCI: 9.23±4.10 CONTROL: 10.22±5.05	37.93	MDA-MCI: 22.86±1.65	MMSE >20, no history of clinical stroke, traumatic brain injury, motor sensory defects, alcohol, or drug abuse/dependence, not diagnosed with any significant medical or psychiatric illnesses, GDS <10.		SSP Correct items (MDA-MCI)	21.76	7.09	44	25.86	6.80	58	MMSE, WAIS vocabulary, memory complaints, CAMCOG (language, attention) CVLT	Age, education, occupational, complexity, vocabulary, frequency of reading, leisure and cultural activities, social participation
						SDA-MCI: 27.00±1.81			SSP Correct items (SDA-MCI)	26.00	6.71	43					
Juncos-Rabadán et al. 2014^44^	Cross sectional	170	MDA-MCI: 71.06±8.36 SDA-MCI: 68.96±8.60 MDNA-MCI: 66.78±8.56 Healthy control: 68.16±8.75	MDA-MCI: 10.18±4.09 SDA-MCI: 9.52±4.08 MDNA-MCI: 7.96±3.77 Healthy control: 9.35±4.62	–	MDA-MCI: 23.40±1.58 SDA-MCI: 27.50±1.50 MDNA-MCI: 24.53±2.35	No prior diagnosis of dementia, psychiatric or neurological disorders, severe illness, deafness or blindness, not receiving chemotherapy, not consumers of substances or alcohol, informant-corroborated memory complaints, performance of 1.5 SDs below age norms on the Spanish version of (CVLT), no significant or minimal impact on activities of daily living assessed by the Lawton and Brody Index, not demented according the NINCDS-ADRDA and DMS-IV criteria. Normal or corrected-to-normal vision and hearing and visual acuity	Scored higher than the cutoff point in memory, general cognitive functioning, and specific cognitive tests, no history of clinical stroke, traumatic brain injury, motor-sensory defects, alcohol or drug abuse/dependence, and no diagnosis of any significant medical or psychiatric illnesses. Normal or corrected-to-normal vision and hearing and visual acuity	SSP length (MDA-MCI)	4.40	0.83	32	4.77	0.67	54	Memory complaints (informant), MMSE, CVLT Language, Attention, calculation, Praxis	Age, education, Visual acuity
									SSP length (SDA-MCI)	4.65	0.80	57					
									SSP length (MDNA-MCI)	4.45	0.83	32					
									SSP total errors (MDA-MCI)	11.93	3.97	32	11.68	3.97			
									SSP total errors (SDA-MCI)	11.60	3.96	57					
									SSP total errors (MDNA-MCI)	11.66	5.25	32					
									SSP time to last response (MDA-MCI)	9063.22	4845.18	32	6429.49	1666.14			
									SSP time to last response (SDA-MCI)	6698.75	2526.20	57					
									SSP time to last response (MDNA-MCI)	7892.75	2674.63	32					
Summers and Saunders et al. 2012^33^	cohort	106	Control: 69.36±5.8 Stable MCI: 71.04±7.1 Progressed MCI:* 73.80±7.9	Control: 13.64±3.1 MCI: 12.55±3.0 Progressed: 14.60±3.5	46.25	–	Memory problems with a history of decline; preserved cognitive functioning; intact activities of daily living; no history of significant medical, neurological, or psychiatric condition; no history of major risk factors for vascular disease; and no history of alcohol abuse, sensory impairment, or impairment to hand mobility	No cognitive complaints or medical history of significance, control group was matched to the mean age level of education of the MCI groups.	SSP length (Stable MCI)	4.84	0.69	25	5.84	-0.75	25	WTAR	Age, education, FSIQ, DRS, sex, RAVLT, BNT
									SSP length (progressed)	4.70	0.82	10					
									SWM total errors (stable MCI)	50.24	18.46	25	32.92	12.95			
									SWM total errors (progressed)	45.90	9.18	10					
									RVP A’(stable MCI)	0.864	0.04	25	0.939	0.04			
									RVP A’ (progressed)	0.820	0.05	10					
Klekociuk and Summers et al. 2014^45^	cohort	118	60–90	–	38.98	–	No previous medical, neurological, or psychological conditions, no evidence of dementia, AEMSS score ≥9, preserved activities of daily living, subclinical impairment as a performance 1.28 standard deviations or greater below age-appropriate normative references.	No previous medical, neurological, or psychological conditions, no evidence of dementia, AEMSS score ≥9, preserved activities of daily living. No evidence of subclinical impairment.	SSP length (a-MCI)	5.55	0.91	22	5.20	0.84	49	RAVLT, DRS-2, FSIQ, digit span, WAIS–III, LNS	Age, education, sex, HADS Depression
									SSP length (na-MCI)	4.72	0.74	25					
									SSP length (a-MCI+)	4.68	0.72	22					
									SWM total errors (a-MCI)	20.73	17.78	22	29.16	18.37			
									SWM total errors (na-MCI)	30.52	17.31	25					
									SWM total errors (a-MCI+)	38.18	17.08	22					
									SWM strategy (a-MCI)	29.64	6.87	22	30.41	6.88			
									SWM strategy (na-MCI)	33.52	5.85	25					
									SWM strategy (a-MCI+)	33.23	6.02	22					
									RVP latency (a-MCI)	407.08	90.50	22	469.34	9.30			
									RVP latency (na-MCI)	542.48	109.6	25					
									RVP latency (a-MCI +)	522.95	155.29	22					
Cacciamani et al. 2018^46^	cohort	25	MCI-AD: 68.58±6.65 MCI-ambiguous: 68.69±7.75	MCI-AD†: 10±3.94 MCI-ambiguous: 9±4.01	40	27.04±0.31	Age between 55 and 80 years, presence of subjective memory complaints, presence of memory impairment as documented by scoring at least 1 SD below cutoff point on the Logical Memory II subscale (delayed recall) from the WMS; Preserved or slightly impaired functional abilities on FAQ, not responding to diagnostic criteria for dementia, MMSE≥24, CDR score of 0.5, GDS <6, Hachinski Modified Ischemic scale ≤4, at least 5 years of formal education		SWM Strategy (overall MCI)	37.44	0.72	25	–			Total-tau, phospho-tau	Age, sex, education, FAQ score, Aβ42, CDR, MMSE, FAQ, GDS, Hachinski Modified Ischemic Scale, Logical Memory II, Mental Deterioration Battery, RAVLT
									SWM strategy (MCI-AD)	38.17	0.95	12					
									SWM strategy (MCI-ambiguous)	36.77	1.06	13					
									SWM errors (overall MCI)	53.96	4.25	25					
									SWM errors (MCI-AD)	59.92	5.73	12					
									SWM errors (MCI-ambiguous)	48.46	6.04	13					
									RVP A’ (overall MCI)	0.81	0.02	25					
									RVP A’ (MCI-AD)	0.81	0.03	12					
									RVP A’ (MCI-ambiguous)	0.82	0.01	12					
Reijs et al. 2017^10^	cohort	263	68.3±9.1	10.4±4.5	56	25.5±3.9	Memory clinic referral for the evaluation of cognitive complaints, age >60 years, a MMSE-score >19, one or more cognitive impairments on neuropsychological tests according to Petersen's criteria, and no clinical diagnosis of dementia.	Age >40, MMSE >the 10th percentile according to age- and education-adjusted local norms, no cognitive impairment on neuropsychological tests	SWM errors	29.6	8.8	73	19.2	10.0	46	Age, sex, education, MMSE, CDR, Aβ42, t-tau, wordlist learning and delayed recall and recognition, animal fluency	FAQ
Klekociuk and Summers et al. 2014^47^	cohort	122	a-MCI: 70.61±7.99 na-MCI: 70.58±5.97 a-MCI+‡: 69.26±6.56 Control: 72.66±6.52	a-MCI: 14.43±3.16 na-MCI: 14.92±3.52 a-MCI+: 12.48±3.53 Control: 14.20±3.74	39.34	–	Presence of cognitive complaints (e.g., memory, attention); preserved general cognition (as assessed by the DRS-2; self-reported capacity to maintain independent daily functioning (confirmed by an informant); no history of major medical, neurological, or psychiatric illness); no history of major risk factors for vascular disease and no history of sensory impairment or impairment to hand mobility.		SWM total errors (a-MCI)	20.29	17.77	23	29.42	18.52	50	WTAR (FSIQ), DRS-2, Digit Span (forward and backward)	Hospital Anxiety and Depression Scale, LNS, Age, education, HADS, Letter-Number Sequencing total, Digit Symbol Coding
									SWM total errors (na-MCI)	32.04	18.83	26					
									SWM total errors (a-MCI+)	36.63	18.96	23					
									SSP length (a-MCI)	5.52	0.91	23	5.19	0.84			
									SSP length (na-MCI)	4.73	0.71	26					
									SSP length (a-MCI+)	4.74	0.71	23					
									RVP latency (a-MCI)	482.92	112.31	23	468.04	89.73			
									RVP latency (na-MCI)	545.36	108.20	26					
									RVP latency (a-MCI+)	519.02	152.07	23					
									RVP A’ (a-MCI)	0.90	0.047	23	0.902	0.042			
									RVP A’ (na-MCI)	0.87	0.05	26					
									RVP A’ (a-MCI+)	0.85	0.047	23					
Saunders and Summers et al. 2011^48^	cohort	106	Control: 69.19±5.75 na-MCI: 71.41±7.22 a-MCI: 70.96±6.85	Control: 13.50±3.09 na-MCI: 13.17±3.50 a-MCI: 13.04±3.39	–	–	Memory problems with a history of decline; preserved cognitive functioning; intact activities of daily living; no history of significant medical, neurological, or psychiatric condition; no history of major risk factors for vascular disease; no history of alcohol abuse, sensory impairment, or impairment to hand mobility		SWM strategy (a-MCI)	37.61	23.56	52	35.42	15.29	26	RAVLT trial 5, DRS-2 AEMSS, RAVLT, GDS, BNT	Age, education, WTAR, FSIQ
									SWM strategy (na-MCI)	38.23	10.71	29					
									SWM errors (a-MCI)	50.50	14.56	52	33.39	12.84			
									SWM total errors (na-MCI)	43.59	14.75	29					
									SSP length (a-MCI)	4.90	0.57	52	5.82	0.76			
									SSP length (na-MCI)	5.22	0.69	29					
									RVP latency (a-MCI)	513.33	126.19	52	445	76.48			
									RVP mean latency (na-MCI)	529.58	125.63	29					
									RVP A’(a-MCI)	0.87	0.07	52	0.93	0.05			
									RVP A’(na-MCI)	0.85	0.05	29					
Stonsaovapak et al. 2020^49^	cohort	45	MCI: 68.39±8.37 Control: 68.39±8.37	–	8.88	–	MCI diagnosis based on the criteria of the MCI Working Group of the European Consortium on Alzheimer's disease, age between 45 and 90 years, with a TMSE score of >23, (MoCA)-Thai score of <25		SWM between errors	53.48	13.10	23	51.45	14.81	22		age, sex, educational, TMSE score, MoCA
									SWM total errors	55.39	13.31		52.64	15.51			
									RVP mean latency	2.77	0.12		2.72	0.11			
									RVP total hits	10.61	5.23		13.68	4.57			Gender, education, CCI
Campos-Magdaleno et al. 2021^50^	cohort	208	Control: 64.26±8.83 MCI-stable: 70.94±7.54 MCI-worsened: 75.44±7.14	Control: 10.28±4.71 MCI-stable: 9.15±3.40 MCI-worsened: 9.30±4.79	35.13	Control: 28.34±1.34 MCI-stable: 25.13±2.89 MCI-worsened: 24.04±2.53	No previous diagnosis of MCI or dementia, clinical stroke, traumatic brain injury, motor-sensory defects, alcohol or drug abuse/dependence, or any neurological or psychiatric disease. Self-reported, informant corroborated concerns about cognition, 1.5 SDs below age and education norms in one or more cognitive domains in CAMCOG-R except for memory, assessed by CVLT, no significant or minimal impact on activities of daily living	No previous diagnosis of neurologic disorders, normal adults in general functioning and specific domain tests, attending primary care health centers with self-reported cognitive concerns; confirmation of these concerns by the short Spanish version of the questionnaire for subjective memory complaints	SSP (MCI-stable)	4.5	2.82	32	5.00	3.84	149	age, Lawton-Brody, SCC, Praxis CAMCOG-R, MMSE, CVLT	
									SSP (MCI-worsened)	3.9	3.27	27					

* In this study, “Progressed” is used for the participants who were classified at baseline as a-MCI or na-MCI but were reclassified as a-MCI following the 20-month assessment

† Subjects with mild cognitive impairment and Alzheimer's disease like CSF profiles

‡ MCI+ defined as multiple domains amnestic mild cognitive impairment in this study; CANTAB: Cambridge Neuropsychological Test Automated Battery; MMSE: Mini-Mental State Examination; WM: working memory; MCI: mild cognitive impairment; SWM: spatial working memory; SSP: spatial span; RVP: rapid visual information processing; DMS: delayed matching to sample; S-MCI: subjective MCI; A-MCI: amnestic MCI; NPIQ: Neuropsychiatric Inventory Questionnaire; FAQ: Functional Activities Questionnaire; APOE: apolipoprotein; Aβ: amyloid-beta; SDA-MCI: single-domain MCI; CFQ: Cognitive Failures Questionnaire; MDA-MCI: multiple-domain amnestic MCI; MNDA-MCI: multiple-domain non-amnestic MCI; GDS: Geriatric Depression Scale; BNT: Boston Naming Test; TMSE: Thai Mental State Examination; MoCA: Montreal Cognitive Assessment; CERAD: Consortium to Establish a Registry for Alzheimer's Disease; CAMCOG: Cambridge Cognitive Examination; CVLT: California Verbal Learning Test; DRS: dementia rating scale; RAVLT: Rey Auditory Verbal Learning Test; RCFT: Rey Complex Figure Test and Recognition Trial; FSIQ: Full Scale Intelligence Quotient; WTAR: Wechsler test of adult reading. CCI: Charlson Comorbidity Index; DSP: Digit Span; WAIS–III: Wechsler Adult Intelligence Scale, 3rd edition; LNS: Letter–Number Sequencing; MMSE: Mini Mental State Examination; CDR: Clinical dementia rating; FAQ: the Functional Assessment Questionnaire; WMS: Wechsler Memory Scale; SMCQ: subjective memory complaints questionnaire; SD: standard deviation, NINCDS-ADRDA: the National Institute of Neurological and Communicative Disorders and Stroke and the Alzheimer's Disease and Related Disorders Association; TMSE: Thai mental state examination; MOANS: Mayo Older American Normative; AEMSS: age- and education-corrected MOANS scaled scores (AEMSS) score.

### Characteristics of the studies and participants

Five of included studies were cross-sectional and nine were cohorts. Only baseline data of the cohort studies are taken into account. In sum, 930 out of 1670 participants were diagnosed with MCI, and 527 were healthy controls. The mean age of the participants was between 55 and 75 years. The years of education varied from 7 to 14, and the male ratio varied between 8 and 56%.

### MCI diagnosis

In this study, most of the researchers used MMSE for the diagnosis of MCI, and the rest of the studies used the other tests or criteria, such as Petersen criteria, MOCA, Rey Auditory Verbal Learning Test, and Dementia Rating Scale.

### CANTAB tests for WM

Regarding the tests for WM in CANTAB, 11 of the included studies reported SWM, and 9 of them reported SSP for assessing WM. As mentioned before, RVP has a small WM component and was used in nine of our included studies. Only one study reported that used delayed matching to sample (DMS) subtest of CANTAB as an assessment tool for WM.

### Factors associated with WM in MCI

In terms of factors associated with WM functions of MCI patients, sex, age, psychiatric disorders such as depression, apolipoprotein 4 (ApoE4), and functional activity scores were not significantly correlated to CANTAB WM scores, while a higher cerebrospinal fluid (CSF) levels of tau-protein and amyloid-beta (Aβ) were associated with a lower function in WM tests.

### Meta-analysis

**Figure 2:**
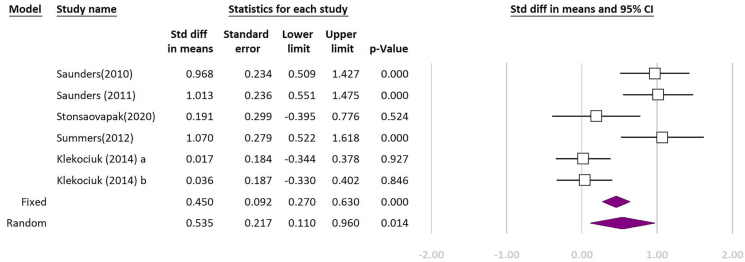
Meta-analysis of comparing patients with mild cognitive impairment and healthy controls based on “total errors” measure of spatial working memory (SWM) test of Cambridge Neuropsychological Test Automated Battery. The purple indicator is the final result. (Test for heterogeneity: I^2^: 81.28%; p<0.01).

**Figure 3:**
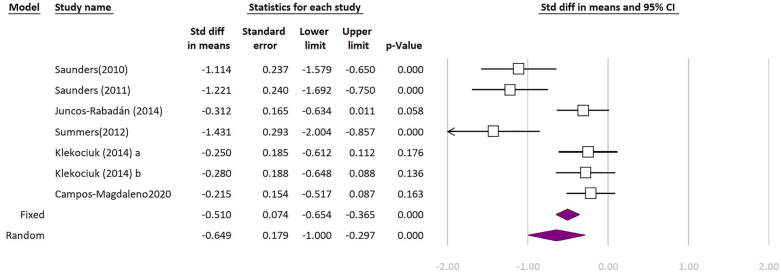
Meta-analysis of comparing patients with mild cognitive impairment and healthy controls based on “length” measure of spatial span (SSP) test of Cambridge Neuropsychological Test Automated Battery. The purple indicator is the final result. (Test for heterogeneity: I^2^: 82.76%; p<0.01).

**Figure 4:**
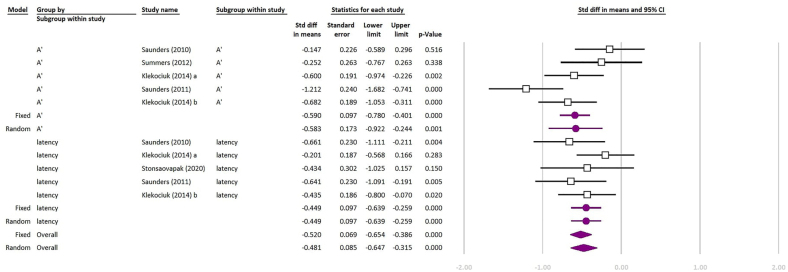
Meta-analysis of comparing patients with mild cognitive impairment and healthy controls based on “A’ and mean latency” measures of Rapid Visual Processing (RVP) test of Cambridge Neuropsychological Test Automated Battery. The purple indicators are the final results. (Test for heterogeneity: overall I^2^: 46.49%; p-value=0.05; A’ I^2^: 67.87 %; p=0.01; Latency I^2^: 0%; p=0.50).

Out of 14 included studies, 3 of them did not include any healthy participants for control group and 4 others did not report our intended component of CANTAB subtests to be included in the quantitative synthesis; hence, they were excluded from the meta-analysis. Seven remaining studies were included in the meta-analysis. The forest plots of the meta-analyses are shown in Figures 2–4. The quantitative synthesis of studies using CANTAB subtests to assess WM showed a significant difference between MCI and healthy controls in SWM (REM SDM: 0.535; 95%CI 0.110–0.960; p=0.014, FEM SDM: 0.450; 95%CI 0.270–0.630; p<0.01; test for heterogeneity I^2^: 81.28%; p<0.01), SSP (REM SDM: 0.649; 95%CI 0.297–1.000; p<0.01, FEM SDM: 0.510 95%CI 0.654–0.365; p<0.01; test for heterogeneity I^2^: 82.76%; p<0.01), and RVP (REM SDM: 0.481; 95%CI 0.316–0.647; p<0.01, FEM SDM: 0.52; 95%CI 0.386–0.654; p<0.01; test for heterogeneity I^2^: 46.49%; p=0.05). Also, RVP A’ (REM SDM: 0.583; 95%CI 0.244–0.922; p<0.01, FEM SDM: 0.590; 95%CI 0.401–0.870; p<0.01; test for heterogeneity I^2^: 67.87%; p=0.01) and RVP latency (REM and FEM SDM: 0.449; 95%CI 0.259–0.639; p<0.01; test for heterogeneity I^2^: 0%; p=0.50) were significantly different between patients with MCI and healthy controls.

### Risk of bias

The results of the RoB assessments are shown in Table 2 ^10,33,39–50^. There was not any exposure studied in our systematic review; so, the third question of the checklist, which assessed the validity of the exposure measurement, was not applicable. Furthermore, we only considered MMSE, Petersen, and MoCA as standard index tests for MCI diagnosis. Because of that, the overall rate of standard measurement of conditions was low. Besides, only 35.7% of the studies mentioned the setting properly. Briefly, there were no considerable levels of bias in most of the included studies.

**Table 2:** Results of risk of bias assessment.

Study	Q1	Q2	Q3	Q4	Q5	Q6	Q7	Q8
Nathan et al., 2017^40^	Yes	No	NA	Yes	Yes	Yes	Yes	Yes
Saunders and Summers, 2010^41^	Yes	No	NA	No	Yes	No	Yes	Yes
Égerházi et al., 2007^42^	No	No	NA	Yes	Yes	Yes	Yes	No
Collie et al., 2002^39^	Yes	No	NA	Yes	Yes	Yes	Yes	Yes
Facal et al., 2014^43^	Yes	No	NA	Yes	Yes	Yes	Yes	Yes
Juncos-Rabadán et al., 2014^44^	Yes	Yes	NA	Yes	Yes	Yes	Yes	Yes
Summers and Saunders, 2012^33^	Yes	No	NA	No	Yes	No	Yes	Yes
Klekociuk and Summers, 2014^45^	Yes	Yes	NA	No	Yes	Yes	Yes	Yes
Cacciamani et al., 2017^46^	Yes	No	NA	Yes	Yes	Yes	Yes	Yes
Reijs et al., 2017^10^	Yes	Yes	NA	Yes	Yes	Yes	Yes	Yes
Klekociuk and Summers, 2014^47^	Yes	Yes	NA	No	Yes	Yes	Yes	Yes
Saunders and Summers, 2011^48^	Yes	No	NA	No	Yes	Yes	Yes	Yes
Stonsaovapak et al. 2020^49^	No	No	NA	No	Yes	Yes	Yes	Yes
Campos-Magdaleno et al., 2020^50^	Yes	Yes	NA	Yes	Yes	Yes	Yes	Yes
Overall	85.7%	35.7%	NA	57.1%	100%	85.7%	100%	92.8%

NA: not applicable.

## DISCUSSION

This study assessed the WM function of patients with MCI and compared it between MCI patients and healthy people using the CANTAB. Also, influencing factors on WM were considered. SWM, SSP, and RVP were the most commonly used subtests of CANTAB for assessing the WM. The results of quantitative synthesis revealed a significant difference between healthy controls and patients with MCI regarding the CANTAB-based WM assessments. Also, the available evidence suggested a significant correlation between CSF levels of tau-protein and Aβ with WM function in patients with MCI.

One of the preclinically deteriorated domains in AD and MCI is WM^21,51,52^. WM comprises a cognitive spectrum from attention allocation to specific stimuli to complex decision-making. Some studies have suggested WM as an early predictor of AD^53^. Regardless of the method of assessment, WM function is found to significantly deteriorate in MCI^24,54^. WM is subdivided into verbal and visual components^55^. Emrani et al. found that the visual component of WM is more sensitive than verbal WM, for distinguishing between MCI patients and healthy older adults^56^. Align with the aforementioned study, our quantitative synthesis reveals that the WM of MCI patients based on SWM, SSP, and RVP is impaired significantly, so it can be suggested as a proper diagnostic evaluation for MCI.

CANTAB is a novel neuropsychological battery for evaluating cognitive state. This battery has shown promising outcomes in the diagnosis of cognitive function in healthy older adults, MCI, AD, or any other possible diseases that may compromise cognition^32,57^. It has several benefits over traditional paper-pencil tests, such as reducing the risk of human error and data noise, recording reaction times precisely, lowering data storing problems, easing task scoring, and having access to normative comparison^32,58^. Also, CANTAB has a non-verbal structure that makes it more convenient for people with different languages^59,60^. Regarding the disadvantages, CANTAB is a time-consuming test, and providing the test instruments, imposes an extra cost to the clinicians, which limits its usage in resource-limited settings. The accuracy of WM tests of CANTAB battery in distinguishing between MCI patients and healthy older adults was studied in our review and CANTAB has shown to be a proper battery for MCI diagnosis.

As a secondary outcome of the study, we assessed related factors with WM function in MCI patients. Aging is one of the confirmed predictors of cognitive decline^61^. Although WM function is found to be affected by age^62^, in most of our included studies, age was not associated with the WM scores of the patients. This may be because most of the participants in our study were older people while there is a need for the participation of patients with a wider age range to survey the age differences.

The relation between CSF biomarkers and cognitive state is one of the interest areas for research. Soldan et al. in a cohort study investigated the performance of cognitively healthy adults on CANTAB-PAL and found that it was associated with CSF p-tau levels^63^. This study suggested that the AD-related CSF biomarker can predict specific cognitive dysfunctions. In our included studies, Aβ and tau-protein were associated biomarkers with WM functions of MCI patients. On the contrary, ApoE4, which is one of the most studied genetic factors associated with human cognition and one of the well-known predictors of AD^64^, was not associated with WM function of MCI patients, as reported in two studies^39,40^.

This study is a novel and unprecedented review of WM assessment of MCI patients with CANTAB. One of the challenges related to this study was that the included studies did not report the sensitivity and specificity of CANTAB for the diagnosis of WM deficits in MCI patients. This should be considered in future studies. The other related limitation was that the included studies used heterogeneous criteria for baseline diagnosis of MCI; thus, the results cannot be generalized to all of the considered populations. Nevertheless, a comprehensive review of available evidence with a systematic approach was the main strength of this study.

This study reveals that WM is an impaired cognitive domain at MCI. Based on our assessment, WM subtests of CANTAB, including SWM, SSP, and RVP, can pinpoint deficits in MCI patients, so CANTAB-based WM assessment can help the clinicians in the diagnosis of MCI. Also, WM functions of MCI patients are associated with some of the AD-associated biomarkers, such as tau-protein and Aβ. There is a need for future well-designed studies on this topic to reach a comprehensive conclusion in terms of both diagnostic accuracies of WM profiles of CANTAB battery and factors that can affect the WM in MCI patients.
